# Acupuncture in treating cardiovascular disease complicated with depression: A systematic review and meta-analysis

**DOI:** 10.3389/fpsyt.2022.1051324

**Published:** 2022-12-01

**Authors:** Lu Lu, Weiming He, Dandan Guan, Yuanyuan Jiang, Guangyong Hu, Feixiang Ma, Li Chen

**Affiliations:** ^1^Liyang Hospital of Chinese Medicine, Changzhou, Jiangsu, China; ^2^The First College of Clinical Medicine, Nanjing University of Chinese Medicine, Nanjing, Jiangsu, China; ^3^Jiangsu Province Hospital of Chinese Medicine, Nanjing, Jiangsu, China; ^4^Jiangsu Province Academy of Traditional Chinese Medicine, Nanjing, Jiangsu, China; ^5^Jiangyin Lingang Hospital, Wuxi, Jiangsu, China; ^6^Yancheng Third People’s Hospital, Yancheng, Jiangsu, China

**Keywords:** acupuncture, cardiovascular disease, depression, systematic review, meta-analysis

## Abstract

**Background:**

Depression is a serious risk for cardiovascular disease (CVD). Improving depression can alleviate cardiac symptoms and improve quality of life. Studies have shown that acupuncture has a positive effect on depression and CVD. This systematic review and meta-analysis will evaluate the efficacy and safety of acupuncture in the treatment of depression complicated with CVD.

**Methods:**

We searched PubMed, Embase, Cochrane Library, Web of Science, CNKI, Wanfang, VIP, and China Biomedical Literature databases. Randomized controlled trials of acupuncture vs. standard care or sham acupuncture or antidepressants were included. The retrieval time is from database construction to 07 April 2022. We used the “risk of bias” tool of Cochrane Collaboration, and the Review Manager (RevMan.) Version 5.4.1 for statistics analysis. Primary outcomes included Hamilton scale for depression (HAMD), self-rating depression scale (SDS), and the effective rate of depression. Secondary outcomes included frequency of angina pectoris and visual analogue scale (VAS) scores for angina pain.

**Results:**

A total of 2,366 studies were screened based on the search strategy. Twelve eligible studies with a total of 1,203 participants have been identified. The result showed that acupuncture reduced the HAMD score [weighted mean difference (WMD): −3.23; 95% confidence interval (CI): −5.38 to −1.09; *P* = 0.003] and the SDS score (WMD: −1.85; 95% CI: −2.14 to −1.56; *P* < 0.00001) in patients with depression complicated with CVD. Acupuncture also improved the effective rate of depression (risk ratio: 1.15; 95% CI: 1.03 to 1.29; *P* = 0.01). The result also showed that acupuncture reduced the attack frequency of angina pectoris (WMD: −4.54; 95% CI: −5.96 to −3.11; *P* < 0.00001) and the VAS score for angina pain (WMD: −0.72; 95% CI: −1.06 to −0.38; *P* < 0.0001). This article reviewed the significant advantages of acupuncture for depression and the superiority of acupuncture over no-intervention therapy, antidepressant therapy, and psychotherapy in reducing angina frequency and pain intensity in patients with CVD.

**Conclusion:**

This systematic review suggested that acupuncture was a good complementary and alternative therapy for CVD complicated with depression. Considering the limitations of the included research literature, it is still necessary to perform multi-center, large-sample, and double-blind high-quality studies to provide higher-level evidence in the later stage.

**Systematic review registration:**

[https://www.crd.york.ac.uk/prospero/], identifier [CRD42022304957].

## Introduction

The prevalence of cardiovascular disease (CVD) is increasing, and the harms related to CVD complicated with depression are becoming increasingly significant. Patients with CVD often experience emotional issues due to repeated illness and a lack of understanding of the development and prognosis of the disease. The prevalence of depression in patients with CVD ranges from 10 to 40%, and the prevalence of depression increases with the severity of heart disease (1). A national study in New York found that participants with persistently elevated depressive symptoms had a 77 percent increase risk of CVD and a 63 percent increase risk of mortality (2). The risk of cardiovascular events is proportional to the severity of depression (3).

Antidepressant treatment in modern medicine includes antidepressant medication, psychotherapy, complementary, alternative, and exercise therapy (4). All of these can ease depressive symptoms and improve the quality of life of patients with CVD. However, a variety of adverse effects, such as sexual dysfunction, weight changes, and gastrointestinal symptoms, often exist along with the use of antidepressants (5). In total, 30–50% of the patients show no-response to antidepressant medication (6). For these reasons, various non-pharmacological approaches, including psychology consulting and complementary and alternative medicine, are increasingly used to treat depression. And acupuncture is one of the most used non-pharmacological treatments.

In traditional Chinese medicine, the main location of CVD complicated with depression is in heart (7). According to Chinese medicine, the heart can “govern blood and vessels,” can promote and regulate the movement of blood throughout the body. At the same time, the heart can “govern the spirit,” master consciousness, thinking, emotions and other activities. If the heart’s qi and blood are sufficient, the pulses will be strong, the blood will be smooth, the human mind will be clear and agile. If the heart’s qi and blood are vacuity, the heart beat will become weak, the blood will be out of order, and the spirit will be listless. This will cause palpitations and shortness of breath, and mental and spiritual abnormalities. The overall concept of “unity of body and spirit” is the basic theory of traditional Chinese medicine (8). Acupuncture therapy is one of the most distinctive Chinese medicine therapies. The operation of acupuncture is to insert needles (usually filigree needles) into the human body at a certain angle under the guidance of traditional Chinese medicine theory. By stimulating the body’s meridians and acupuncture points, it can play a role in regulating the balance of qi, blood, yin, and yang in the five internal organs, so as to achieve the effect of disease prevention and treatment. Its treatment process requires “governing the spirit” and “protecting the spirit,” which itself is a process of physical and mental harmony.

With the general recognition in the world, acupuncture has gradually become an important alternative therapy for depression (9, 10). Many studies have shown that acupuncture is effective in treating CVD (11). Yet, the efficacy of acupuncture for CVD-related depression is unclear. We conducted this meta-analysis to investigate the efficacy and safety of acupuncture in treating depression complicated with CVD.

## Materials and methods

This study followed the preferred reporting items for systematic reviews and meta-analyses (PRISMA) (12).

### Eligibility criteria

In this review, studies adhering to the following criteria were considered: (1) the study should be a randomized controlled trial (RCT) with or without blinding; (2) the manuscript was written in Chinese or English; (3) the study participants should be diagnosed with CVD, including unstable angina, non-ST-segment elevation myocardial infarction, ST-segment elevation myocardial infarction, stable angina, and ischemic cardiomyopathy; (4) trial interventions must have acupuncture (must be inserted into acupoints or Ashi points), and could include manual acupuncture (MA) and electroacupuncture (EA). Control trials may be sham acupuncture, standard care, antidepressants, and so on; (5) outcome measures in the trial must include an assessment of depression, like Clinical effective rate of depression, Hamilton scale for depression (HAMD), self-rating depression scale (SDS).

Studies belonging to the following categories were excluded: duplicate studies (same patient data published by the same author in different journals); intervention or outcome information unclear or inappropriate; lack of important information on participant characteristics; not available full text; incomplete data.

### Search methods

To perform a systematic review and meta-analysis, our group searched English and Chinese databases from inception to 07 April 2022: PubMed, Embase, Cochrane Library, Web of Science, CNKI, Wanfang, VIP, and China Biomedical Literature databases. The retrieval method adopts the combination of subject headings and free-text terms. Taking PubMed as an example, the subject headings were “coronary disease,” “coronary artery disease,” “acute coronary syndrome,” “myocardial ischemia,” “myocardial infarction,” “angina pectoris,” “coronary vessels,” “percutaneous coronary intervention,” “depression,” “acupuncture,” “needles,” “dry needling”; the free text terms were “coronary heart disease,” “acute coronary syndrome,” “angina,” “ischemic cardiomyopathy,” “myocardial ischemia,” “myocardial infarction,” “coronary,” “percutaneous coronary intervention,” “depress*,” “acupuncture,” “needle,” “needling” (Supplementary Data Sheet 1).

### Selection of studies

Endnote X9 was used to manage the retrieved literature. Two reviewers (GH and DG) independently reviewed the titles and abstracts of the selected references and included or excluded studies based on the eligibility criteria. To make the final selection, we subscribed to the full text of the selected references. Any disagreement in the procedure was addressed by a third reviewer (LL) through discussion or arbitration. The reasons for exclusion were carefully documented.

### Data extraction and management

After screening the final eligible studies, the data would be extracted independently by two reviewers (FM and YJ). The following information would be extracted: initial author’s name, year of publication, number of participants, age, gender, acupuncture details (e.g., stimulation modality, duration, and frequency), control group, and outcome. The corresponding author would be contacted if any relevant data is missing. Any disagreement between the two reviewers would be resolved by discussion, and further disagreements would be arbitrated by the third reviewer (LC).

### Assessment for risk of bias

Two reviewers (LL and FM) conducted an independent assessment of the risk of bias in the final selected studies using the “risk of bias” tool of Cochrane Collaboration. This tool consists of seven items, which are selection bias (random sequence generation and allocation concealment), performance bias (blinding of participants and personnel), detection bias (blinding of outcome assessment), attrition bias (incomplete outcome data), reporting bias (selective outcome reporting), and other sources of bias. Each study was evaluated as high, low, or unclear risk of bias in all domains, and the assessment criteria were based on the Cochrane handbook. Disagreement between the two reviewers was settled by discussion.

### Statistical analysis

Statistics analysis was done using the Review Manager (RevMan). Version 5.4.1, The Cochrane Collaboration 2020.

All the studies were grouped and evaluated according to their outcome variables and intervention characteristics. For continuous data, the weighted mean difference (WMD) was used, and for dichotomous data, the risk ratio (RR) was used. If the trials gave only the mean and standard deviation (SD) before and after treatment, we estimated the change in mean and SD using the methods described in Cochrane handbook v6.3, chapter 16.1.3.2.

In the analysis, we chose the 95% confidence interval (CI). Due to the diversity of acupuncture treatments and methods, statistical heterogeneity is almost unavoidable. Even with needling, there are differences in acupoint selection, acupoint allocation, acupuncture depth, acupuncture manipulation, needle retention time, treatment frequency, total treatment times, and acupuncturist qualification. So a random-effects model was used regardless of whether *I*^2^ was > 50% or not. We used subgroup and sensitivity analyses to identify the causes of the significant heterogeneity.

## Results

### Description of included studies

**FIGURE 1:**
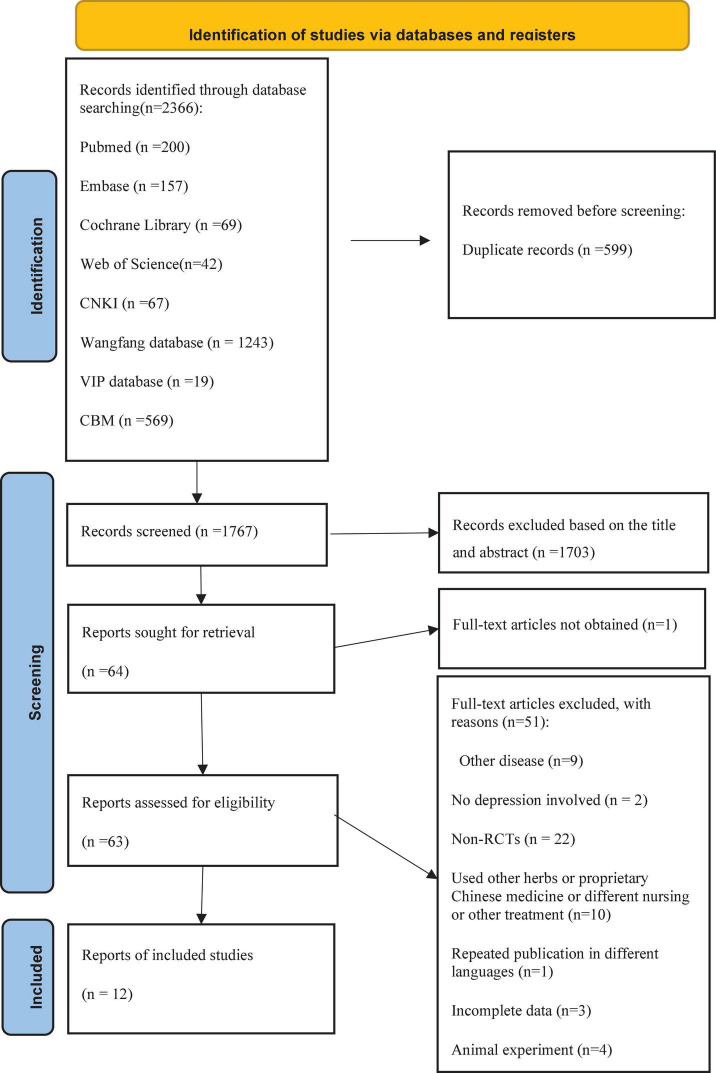
Flow chart of the study selection process.

A total of 2,366 studies were identified by both electronic searches and cited references. Of these, 599 articles were duplicated, 1,703 articles were excluded for inappropriate titles or abstracts, and the data of one article was incomplete. After further screening, a total of 12 studies (13–24) satisfied the criteria. A flow chart of the study selection process was shown in Figure 1. Of the 12 studies, seven studies were postgraduate candidate thesis (14–16, 19–21, 24), and the remaining were journal articles. All studies were conducted in China. The 12 studies were all published between 2007 and 2021. Five were multi-center studies (14, 16, 19, 20, 23), and the rest were single-center studies. The total number of subjects of the individual studies varied from 28 to 398, with a total of 1,203 participants, including 569 males and 634 females. All acupuncture interventions were based on the underlying treatment of CVD. Regarding the stimulation of acupuncture, eight trials chose MA (16–22, 24), and four trials chose EA (13–15, 23). A description of the characteristics of the included studies can be found in Table 1.

**TABLE 1:** Characteristics of included studies.

	Design (interventions)		Number					
							
References	EG	CG	EG/CG	Mean age (Mean ± SD)/Age range	Acupuncture points (EG)	Treatment duration (day)	Outcome measure	Frequency and retention time
Bai et al. (13)	Acupuncture	Flupentixol melitracen	38/38	EG: 58.27 ± 7.16 CG: 59.35 ± 6.07	BL15, BL17, BL23, DU26, RN17, PC6, PC4, ST36*, SP6*	20	HAMD-17	Once/day, 20 min
Fan (14)	EG1: Acupuncture (i) EG2: Acupuncture (ii) EG3: Needle (iii)	No intervention	91/93/97/93	EG1: 61.98 ± 9.6919 EG2: 61.42 ± 9.786 EG3: 62.48 ± 8.990 CG: 63.59 ± 10.119	EG1: PC6*, HT5* EG2: LU6*, LU9*	28	SDS, attack frequency of angina pectoris, VAS	3 times/week, 30 min
Liu et al. (17)	Acupuncture	No intervention	46/52	EG: 71 ± 10.23 CG: 70 ± 10.56	ST36	56	HAMD-24	Twice/day, 30 min
Liu (18)	Acupuncture	Fluoxetine hydrochloride	30/30	EG: 45.6 CG: 44.5	DU20, RN17	45	The clinical effective rate of depression	Once/day, 45 min
Liu (19)	Acupuncture and Sertraline	Sertraline	36/36	EG: 58.03 ± 7.23 CG: 57.31 ± 8.23	DU20, DU29, HT7, PC6, LR3, ST40, SP6	28	HAMD-24, Clinical effective rate of depression	5 times/week, 20 min
Lv (20)	EG1: Acupuncture (i) EG2: Needle (iii)	No intervention	79/82/82	EG1: 67.76 ± 8.36 EG2: 63.06 ± 8.49 CG: 64.69 ± 9.86	EG1: PC6, HT5	28	Attack frequency of angina pectoris, SDS	3 times/week, 30 min
Sun (21)	Acupuncture	Sertraline	30/30	EG: 60.00 ± 9.54 CG: 59.80 ± 7.36	BL13, BL15, BL17, BL18, BL20, BL23	56	HAMD-17, SDS, Clinical effective rate of depression	3 times/week, 30 min
Sun et al. (22)	Acupuncture	No intervention	27/26	EG: 63.78 ± 13.37 CG: 58.12 ± 12.10	LR13, LR14, LR3	7	SDS	Once/day, 30 min
Wang et al. (23)	Acupuncture	No intervention	15/15	EG: 55–66 CG: 53–68	PC6*	28	Attack frequency of angina pectoris, VAS, SDS	3 times/week, 30 min
Gao (15)	Acupuncture (iv)	No intervention	60/60	EG: 59.4 ± 11.9 CG: 60.0 ± 11.5	DU20, DU29, LR3, HT7, PC6, RN17, iv*	84	HAMD-24	5 times/week, 20 min
Zhang (24)	Acupuncture	Psychotherapy	22/23	EG: 65 ± 9.3 CG: 66 ± 8.7	LI4, LR3, DU20, DU29	42	The clinical effective rate of depression	5 times/week, 30 min
Lan (16)	Acupuncture (i)	Acupuncture (ii)	14/14	EG: 64.85 ± 6.03 CG: 65.78 ± 7.74	EG: PC6, HT5 CG: LI5, LI6	14	Attack frequency of angina pectoris, VAS, SDS	5 times/week, 30 min

CG, control group; EG, experimental group; i, acupoints on disease affected meridian; ii, acupoints on non-affected meridian; iii, non-acupoints; iv, dialectical acupoint based on the theory of “the Midnight-Noon Ebb-Flow”; * electro-acupuncture.

Regarding the diagnosis of CVD, three of the included reports were after percutaneous coronary intervention (13, 15, 19), four were stable angina pectoris (14, 16, 20, 23), and three were acute coronary syndrome (17, 22, 24), and two were only described as coronary heart disease (18, 21). Of all the included reports, six of them affirmed a diagnosis of depression in their inclusion criteria, and the HAMD ranged from 8 to 35 (13, 15, 17, 19, 21, 24). The remaining six reports used the improvement of depression as the efficacy evaluation index (14, 16, 18, 20, 22, 23).

Of the 12 included RCTs, nine studies used a two-arm parallel design, and four of which compared acupuncture with antidepressants [flupentixol melitracen (11), sertraline (19, 21), and fluoxetine hydrochloride (18)],while the remaining studies compared acupuncture on disease affected meridian with acupuncture on non-affected meridians (16), and acupuncture with no intervention (15, 17, 22) or psychotherapy (24). A four-arm parallel study (14) compared the effects of four groups: disease-affected meridian acupuncture, non-affected meridian acupuncture, sham acupuncture, and alternate control. One three-arm parallel study (20) evaluated meridian acupuncture, sham acupuncture, and alternate control. In a three-arm parallel study (23), one control group was selected of healthy individuals. This did not meet the inclusion criteria and was excluded. The other two groups that included patients with CVD treated with acupuncture or no intervention were included in the analysis.

### Methodological quality of included studies

**FIGURE 2:**
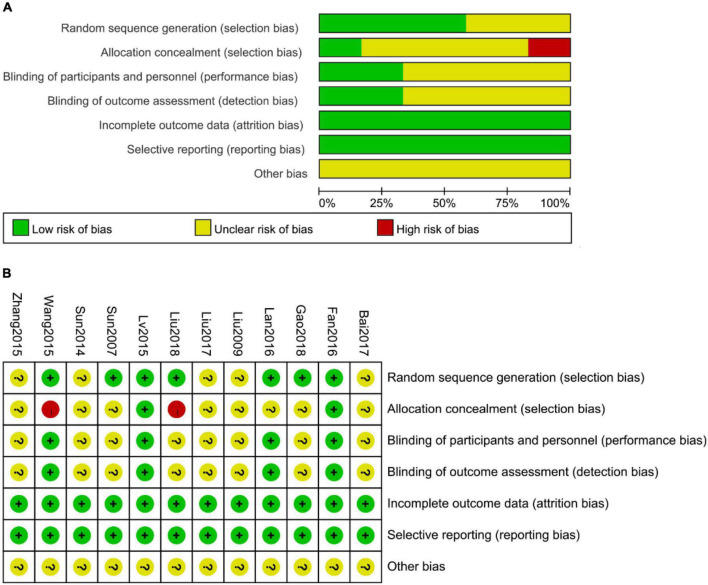
The risk of bias assessment with the Cochrane Collaboration tool. **(A)** Risk of bias graph. **(B)** Risk of bias summary.

The risk of bias assessment in the studies is shown in Figure 2. Random sequence creation, allocation concealment, participant blinding, personnel and outcome assessment, insufficient outcome data, selective outcome reporting, and similar baseline characteristics were all identified at risk for bias. Although all of the studies involved randomization, only seven of them discussed random sequence creation methods in detail (12–16, 19–21, 23). Two of them (14, 20) used the central randomization system to reduce selection bias, two (19, 23) used a spread-out random allocation table, and the rest did not mention allocation concealment. Four studies (14, 16, 20, 23) described the blinding of patients, but the other studies did not describe the details. In these four literature reports, researchers, operators, and statisticians were separated and others did not describe the details of blinding for outcome assessment. According to the authors, baseline characteristics (age, sex, race, and disease duration) of patients in the different groups were similar in the studies.

### Primary outcomes

#### Hamilton scale for depression score

**FIGURE 3:**
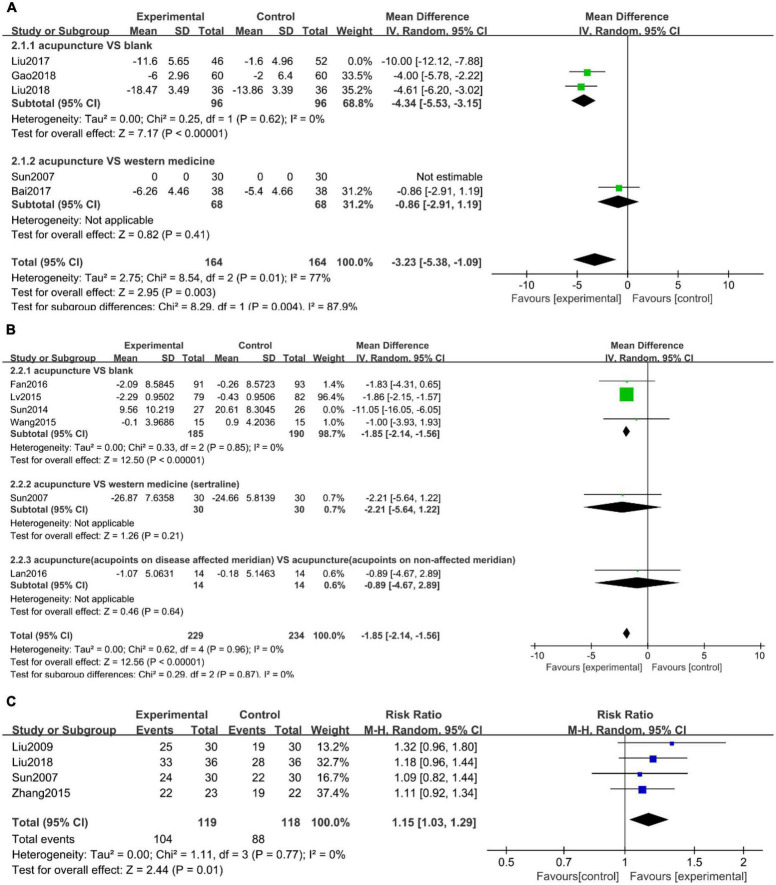
Forest plots of **(A)** Hamilton depression rating scale (HAMD) scores, **(B)** self-rating depression scale (SDS) scores, and **(C)** clinical effective rate.

Of the five studies (13, 15, 17, 19, 21) that used the HAMD score, Sun (21) was excluded due to incomplete data. Subgroup analysis was carried out according to the type of control intervention, such as no intervention and western medicine. A sensitivity analysis revealed that Liu et al. (17) study was the primary source of heterogeneity in the subgroup analysis (Supplementary Data Sheet 2). After subgroup analysis and excluding the study in question, the heterogeneity was reduced. The result showed that acupuncture was more effective (Figure 3A, WMD: −3.23; 95% CI: −5.38 to −1.09; *P* = 0.003).

#### Self-rating depression scale score

There were six studies (14, 16, 20–23) that measured the SDS. According to the type of control intervention, we carried out subgroup analysis, such as no intervention, western medicine, and acupoints on the non-affected meridian. The result showed that acupuncture was more effective compared to no intervention (Figure 3B, WMD: −1.85; 95% CI: −2.14 to −1.56; *P* < 0.00001).

#### Clinical effective rate of depression

Clinical effective rate of depression was reported in four studies (18, 19, 21, 24) with low heterogeneity (Figure 3C, *I*^2^ = 0%, *P* = 0.77). Considering clinical heterogeneity, a random-effects model was still used. Meta-analysis showed that the effective rate of the acupuncture treatment group was statistically different from that of the control group (Figure 3C, RR: 1.15; 95% CI: 1.03 to 1.29; *P* = 0.01).

### Secondary outcomes

#### Attack frequency of angina pectoris

**FIGURE 4:**
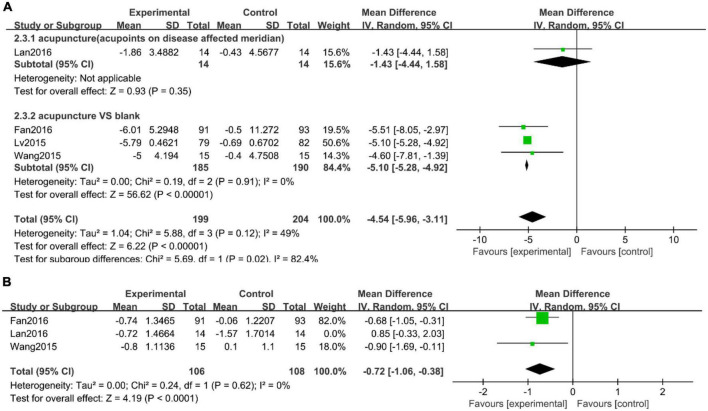
Forest plots of **(A)** attack frequency of angina pectoris, **(B)** visual analogue scale (VAS).

Four studies (14, 16, 20, 23) with heterogeneity (Figure 4A, *I*^2^ = 49%, *P* = 0.12) noted the attack frequency of angina pectoris. Due to the different types of control interventions, we conducted a subgroup analysis, divided into no intervention, acupoints on the non-affected meridian. The results showed that acupuncture therapy was more effective than the control group in the attack frequency of angina pectoris (Figure 4A, WMD: −4.54; 95% CI: −5.96 to −3.11; *P* < 0.00001).

### Angina pain intensity

Three studies (14, 16, 23) used the Visual analogue scale (VAS) as an assessment of angina pain intensity. VAS was 10 cm in range (0 means no pain, 10 means worst pain). Due to the high heterogeneity of the three studies, we used a sensitivity analysis to find that Lan (16) was the main source of heterogeneity (Supplementary Data Sheet 2). Our results indicated that acupuncture was more effective at reducing pain (Figure 4B, WMD: −0.72; 95% CI: −1.06 to −0.38; *P* < 0.0001).

### Assessment of adverse events

**FIGURE 5:**
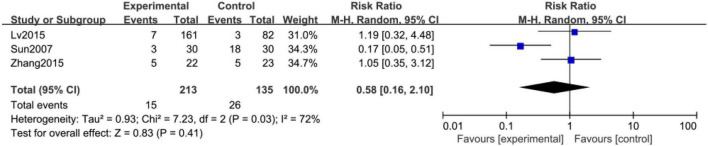
Forest plot of safety profile and adverse events of acupuncture-treated CVD complicated with depression.

Nine studies (13–16, 19–22, 24) reported adverse events. Four of them (13, 15, 19, 22) reported no adverse events occurred. Fan (14) reported one serious adverse event of death from acute myocardial infarction in a patient who was not treated with acupuncture. The study also reported 16 cases of adverse reactions, eight cases had allergic reactions due to electrode allergy, which was not related to the treatment method. And the remaining eight cases had adverse effects such as subcutaneous bleeding, acupuncture pain, and insomnia. The intervention methods in Lan (16) were all needling, and there were four cases of adverse reactions, one case of dizziness, and three cases of local bruising from acupuncture. Another three studies showed adverse events in both groups. The adverse events in experimental groups included subcutaneous hemorrhage, pain or numbness from acupuncture, rapid heartbeat, dizziness, diarrhea, nausea, insomnia, and fainting from acupuncture. The authors, however, said no serious incidents occurred, and most were relieved after rest. A meta-analysis of the three studies in Figure 5 shows that there was no significant difference between the two groups (*P* = 0.41).

## Discussion

Cardiovascular disease is one of the factors that affects human health and quality of life around the world, and its occurrence is often accompanied by depression and other negative emotions. When CVD and psychological disorders coexist, they are referred to as psycho-cardiology disease (25). Negative emotions may trigger or exacerbate the symptoms of CVD. Globally there were almost four million estimated ischemic heart disease (IHD) disability-adjusted life years, which can be attributed to major depression in 2010 (26). Depression also increases adverse cardiovascular events, such as myocardial infarction and heart failure. In addition, CVD can lead to mental health problems. The prevalence of depression in various heart diseases is significantly higher than expected in the general health population (27). The pathogenesis of psycho-cardiology disease has not been fully clarified and may be related to hypothalamic-pituitary-adrenal axis (HPA), reduced heart rate variability (28), altered platelet function (29), and increases proinflammatory processes (30). There is considerable functional overlap and crossover between these systems in regulating both cardiac and neuropsychiatric functioning.

As a world-renowned ancient Chinese medical therapy, acupuncture has served the Chinese medical system for over 3,000 years. Acupuncture focuses on the integrity of the human body, the unity of form and spirit. The theory of “healing spirit” is used throughout the treatment process, and the therapeutic effect is achieved by regulating the body’s qi, blood, yin, and yang. A large body of evidence suggests that acupuncture has satisfactory efficacy and safety in the treatment of depression (6, 31, 32) and CVD (33–35). Experiments in animal models of depression have shown that acupuncture increases hippocampal and neuroplasticity, reduces brain inflammation, and has effects on the HPA axis, neuropeptides, and neurotransmitters (34, 36). Acupuncture can also improve autonomic function, increase heart rate variability (37), and participate in immune regulation and inflammation control (38). These may be the mechanisms by which acupuncture improves psycho-cardiology disease.

### Discussion of main results

The proposal of “psycho-cardiology disease” makes people pay more attention to mental health while treating heart disease. Many clinical studies have shown that acupuncture is good at relieving depression and improving cardiovascular symptoms, but it is unclear whether acupuncture can affect psycho-cardiology disease. Therefore, we designed this study to investigate the efficacy and safety of acupuncture in the treatment of depression complicated with CVD. Pooled data showed that acupuncture was more effective than no intervention in improving depression in patients with coronary heart disease. In addition, acupuncture also has a significant effect on reducing the frequency of angina pectoris and the pain intensity of angina pectoris. There were no serious adverse events of acupuncture in all included studies, and the adverse reactions related to acupuncture were relieved by rest.

A total of 27 different acupoints were used in the 11 RCTs, mainly involving pericardium channel of hand-Jueyin (PC), Du channel (DU), heart channel of hand-Shaoyin (HT), Ren channel (RN). The top five acupoints were Neiguan (PC6), Baihui (DU20), Yintang (DU29), Tongli (HT5), Danzhong (RN17). Chinese medical theory emphasizes that “heart governs the blood and vessels” and “heart governs the spirit.” The syndrome of heart disease is mainly manifested by vascular dysfunction and mental abnormalities. Therefore, the treatment of psycho-cardiology disease mainly selects PC and HT. Depression belongs to the category of “depression pattern,” and the disease is located in the brain, which is considered by Chinese medicine as “brain is the house of the spirit.” DU travels through the brain, which can treat brain-related mental illnesses. PC6 is commonly used to treat heart disease and mental disease. Plenty of evidence shows that PC6 can regulate autonomic function, reduce inflammation and stabilize coronary plaque (37). DU20 and DU29 are widely used in the treatment of depression (39). HT5 is often used in the treatment of CVD, with the improvement of heart rate variability, myocardial systolic function, and myocardial blood supply (40). RN17 is the alarm point of the pericardium and is commonly used in the treatment of heart disease and depression (41).

Sensitivity analysis found that Liu et al. (17) and Lan (16) may be the main sources of heterogeneity. After subgroup analysis and exclusion of studies, heterogeneity was effectively reduced. We checked all included studies carefully and found differences between these two studies and the other groups. In Liu et al. (17), only ST36 was selected for acupuncture points, and the frequency of acupuncture was twice a day. In Lan (16), the control group was still acupuncture on the meridians, but not on the affected meridians.

### Strengths and limitations

The result of this systematic review suggests that for CVD-related depression, acupuncture is a good complementary and alternative therapy under the premise of treating CVD. In this review, acupuncture of CVD patients is better than the control group (no intervention control, antidepressant control) in reducing the frequency of angina pectoris and reducing the pain intensity of angina pectoris. In terms of improving depression, the efficacy of acupuncture interventions was inconsistent in different subgroups. Acupuncture was significantly better than no acupuncture for depression, but there was insufficient support that acupuncture was better than antidepressants in the treatment of depression. Since less than 10 articles were included in each analysis, publication bias could not be described with a funnel plot.

Most of the literature has the following methodological problems: (i) the sample size of most experiments is small, which can easily lead to false-positive results; (ii) in terms of acupuncture intervention, the diversification, and individualization of acupoint selection and manipulation are the main features. All kinds of acupuncture treatments are lumped together without distinction. This is an inherent contradiction that is difficult to ignore in this type of systematic review. When determining the treatment plan for the patient, the clinician also needs to make decisions based on clinical knowledge. (iii) The randomization scheme is not clearly described. The random allocation method in most pieces of literature is described as the random number table method, and there is no detailed description of the random scheme. (iv) The application of the blind method is lacking in many studies. Considering the limitations of the included research literature, it is still necessary to collect multi-center, large-sample, and double-blind high-quality RCT studies to provide higher-level evidence in the later stage.

## Data availability statement

The original contributions presented in this study are included in the article/Supplementary material, further inquiries can be directed to the corresponding author.

## Author contributions

LL and LC conceived the study. LC, WH, and LL provided general guidance to draft the protocol. GH and DG designed the search strategy. LL drafted the manuscript. LL, DG, YJ, GH, FM, WH, and LC reviewed and revised the manuscript. All the authors have read and approved the final version of the manuscript.
