# Supplementary material for: Acupuncture in treating cardiovascular disease complicated with depression: A systematic review and meta-analysis
Source: Front Psychiatry. 2022 Dec 1;13:1051324. doi: 10.3389/fpsyt.2022.1051324 (PMC9752033; doi:10.3389/fpsyt.2022.1051324)

Search strategy in PubMed

| NO. | Search items |
| --- | --- |
| #1 | (“Coronary disease”[Mesh]) OR (“Coronary Artery disease” [Mesh]) OR(“Acute Coronary Syndrome” [Mesh]) OR(“Myocardial Infarction” [Mesh]) OR (“Myocardial Ischemia” [Mesh]) OR (“Angina Pectoris” [Mesh]) OR (“Percutaneous Coronary Intervention” [Mesh]) OR (“Coronary Vessels” [Mesh]) |
| #2 | “Coronary heart disease” |
| #3 | “Coronary disease” |
| #4 | “Acute Coronary Syndrome” |
| #5 | “Myocardial Infarction” |
| #6 | “Myocardial Ischemia” |
| #7 | “Ischemia cardiomyopathy” |
| #8 | Angina |
| #9 | “Percutaneous Coronary Intervention” |
| #10 | Coronary |
| #11 | #1 OR #2 OR #3 OR #4 OR #5 OR #6 OR #7 OR #8 OR #9 OR #10 |
| #12 | (“Depression” [Mesh]) |
| #13 | Depress* |
| #14 | #12 OR #13 |
| #15 | Acupuncture |
| #16 | Needl* |
| #17 | (“Acupuncture” [Mesh]) OR (“Needles” [Mesh]) OR (“Dry Needling” [Mesh]) |
| #18 | #15 OR #16 OR #17 |
| #19 | #11 AND #14 AND 17 |


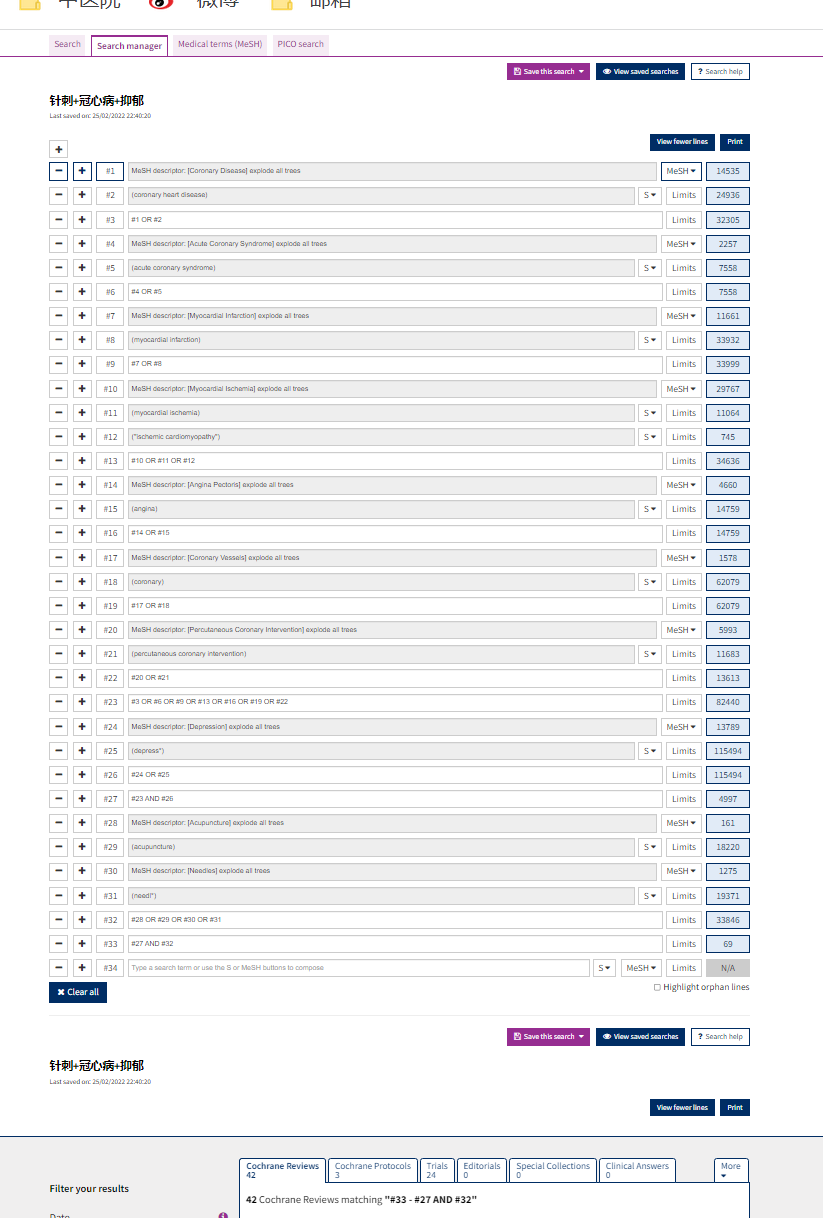

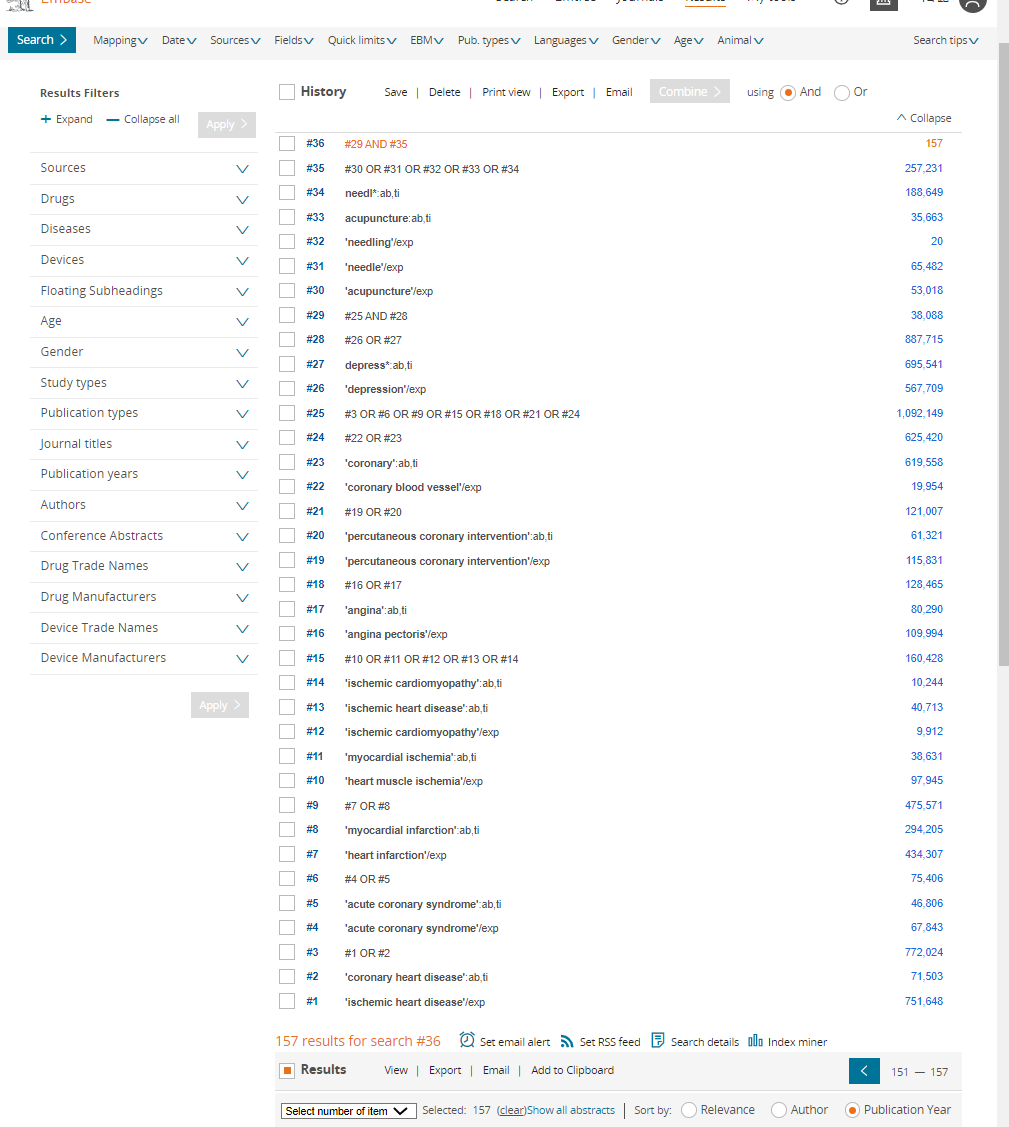

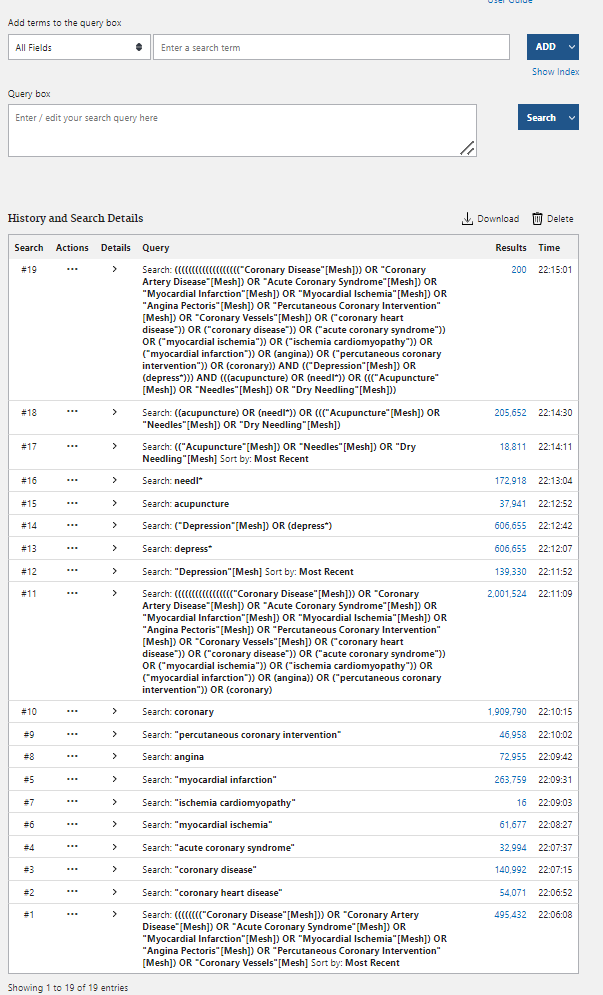

Supplement: Supplementary file 1 [file Data_Sheet_1.docx]
