# Supplementary figures and images for: Acupuncture in treating cardiovascular disease complicated with depression: A systematic review and meta-analysis
Source: Front Psychiatry. 2022 Dec 1;13:1051324. doi: 10.3389/fpsyt.2022.1051324 (PMC9752033; doi:10.3389/fpsyt.2022.1051324)

The results of the two sensitivity analyses in the article are presented below.


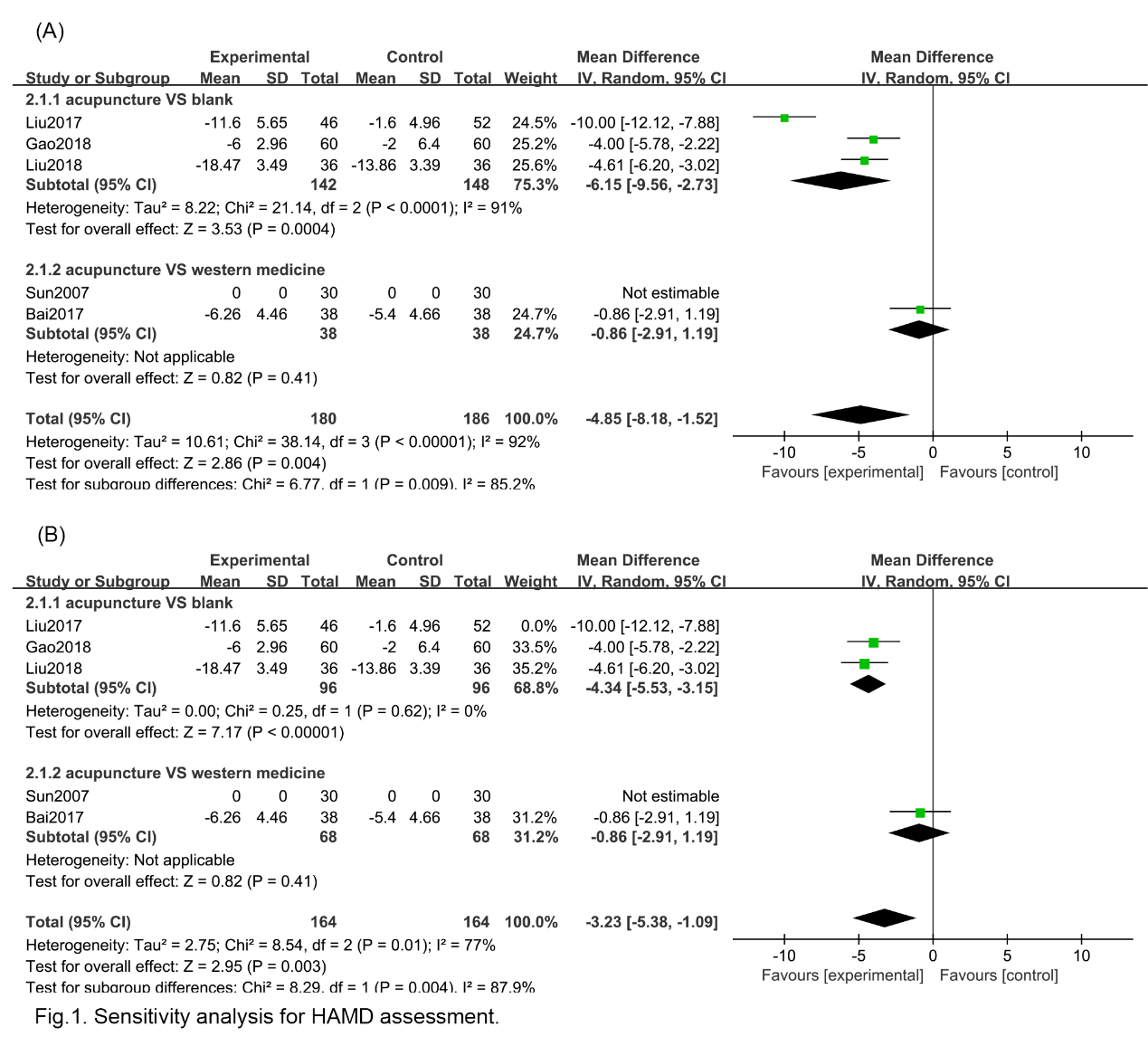


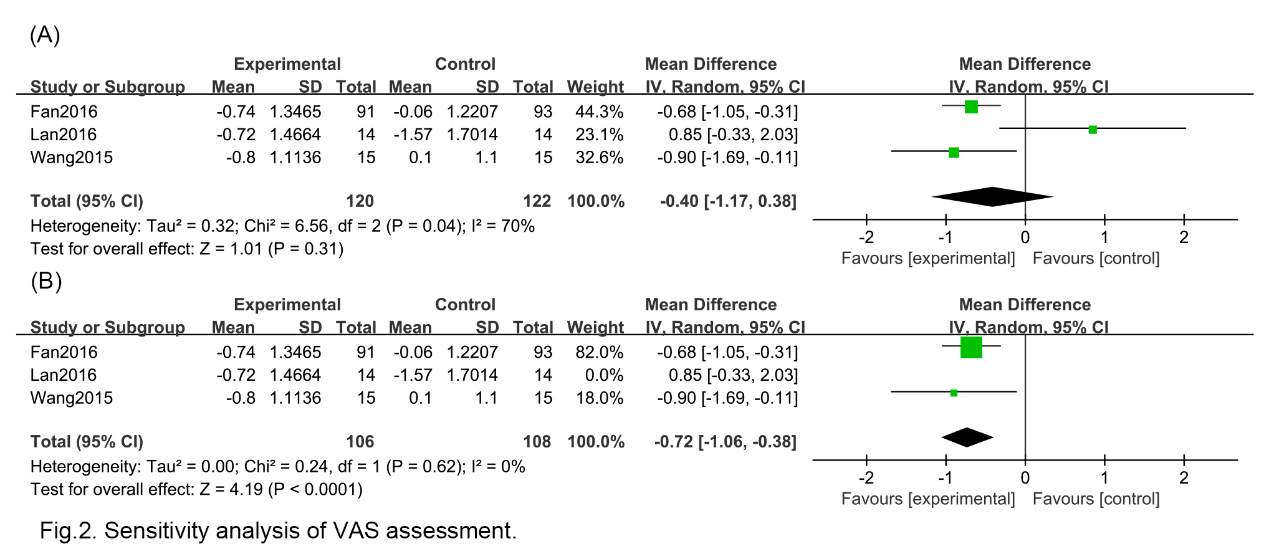

Supplement: Supplementary file 2 [file Data_Sheet_2.docx]
